# Body mass index across adulthood, weight gain and cancer risk: a population-based cohort study

**DOI:** 10.1186/s12885-025-13855-0

**Published:** 2025-03-17

**Authors:** Marko Mandic, Fatemeh Safizadeh, Ben Schöttker, Bernd Holleczek, Michael Hoffmeister, Hermann Brenner

**Affiliations:** 1 Division of Clinical Epidemiology and Aging Research, German Cancer Research Center (DKFZ), Im Neuenheimer Feld 581, 69120 Heidelberg, Germany; 2 Medical Faculty Heidelberg, Heidelberg University, Heidelberg, Germany; 3 Saarland Cancer Registry, Saarbrücken, Germany; 4 German Cancer Consortium (DKTK), German Cancer Research Center (DKFZ), Heidelberg, Germany

**Keywords:** Overweight, Obesity, Cancer, Weight gain, Colorectal cancer, Breast cancer, Excess weight

## Abstract

**Background:**

Although the association between excess weight and cancer risk is well established, it is not known how this association evolves across the lifespan. We aimed to investigate the strength of the association of excess weight at different ages in adulthood and adult weight gain with cancer risk.

**Methods:**

We used data from a German population-based cohort study of 9,218 participants aged 50–75 (mean 62) years recruited between 2000 and 2002. Participants provided socio-demographic, medical, and lifestyle data, including self-reported current height and weight (at ages 20, 30, 40, 50 and baseline). Main exposures were body mass index (BMI, kg/m^2^) at different ages and weight change (kg) since age 20. The outcome was obesity-related cancer (13 types). Hazard ratios (HRs) and 95% confidence intervals (CIs) were estimated using multivariable Cox models.

**Results:**

During a median follow-up of 17.1 years, 852 diagnoses of obesity-related cancers were recorded. Overweight and obesity in early and middle adulthood showed no significant associations with obesity-related cancer risk, whereas significant positive associations were observed for overweight and obesity at age 50 years and older. For weight change since age 20, strong associations were found, with HRs (95% CI) of 1.42 (1.11–1.81), 1.57 (1.24–1.99) and 1.96 (1.56–2.47) for the 2nd, 3rd, and 4th quartile compared to the lowest quartile, respectively. After mutual adjustment for adult weight gain and BMI at baseline, the estimates for weight gain persisted, while those for BMI at baseline disappeared. The main limitation of the study is that the weights were self-reported.

**Conclusions:**

Our findings suggest that excess weight may have a varying effect on cancer risk through life with its impact potentially being more pronounced in later adulthood, and that adulthood weight gain might be a better indicator of obesity-related cancer risk than BMI measured at a single point in time.

**Supplementary Information:**

The online version contains supplementary material available at 10.1186/s12885-025-13855-0.

## Introduction

In 2016, the International Agency of Research on Cancer (IARC) working group published a report stating that there is sufficient evidence for the causal link between body fatness and at least 13 cancer types [1]. Nevertheless, the overweight and obesity rates continue to rise in many parts of the world. Between 1990 and 2022, adult obesity rates have doubled, while adolescent obesity rates have quadrupled. In absolute numbers, this means that approximately one in eight adults worldwide has obesity [2]. These alarming trends emphasize the urgent need for better implementation of public health strategies to reduce overweight and obesity prevalence, which could help in preventing cancers associated with excess weight.

While the relationship between excess weight and cancer has been well established [3], little research has been done to examine the temporal patterns of association between excess body fatness and cancer risk. Most of the epidemiological evidence so far is based on singular measurements of body mass index (BMI), which may not capture the full extent of the relationship. A number of studies have looked at measures of cumulative exposure to excess weight (such as overweight-years, which are analogous to pack-years in smoking) and have found robust associations with several cancer types [4–7]. Nonetheless, these studies have operated under the assumption that excess weight has consistent detrimental effects on cancer risk across all ages. On the other hand, a few studies looking at the life course changes of body weight or body size have also suggested that weight trajectories and weight gain may be better predictors of cancer risk than weight measurements at a single point in time [8, 9]. Overall, it is still unclear how exposure to excess adiposity at different ages is associated with cancer risk.

Using data from a German population-based prospective cohort study with a long follow-up and detailed information on weight during adulthood, we aimed to investigate the strength of the association between excess weight at different ages, adult weight gain and the risk of obesity-related cancer.

## Methods

### Study design and population

This study is based on data from the ESTHER study (German - *Epidemiologische Studie zu Chancen der Verhütung*,* Früherkennung und optimierten Therapie chronischer Erkrankungen in der älteren Bevölkerung*), a statewide population-based prospective cohort study conducted in Saarland, Germany. Details of the study design have been reported elsewhere [10]. In brief, between 2000 and 2002, 9,940 men and women aged 50–75 years were recruited by their general practitioners (GPs) during routine health check-ups. The baseline socio-demographic characteristics of the study population were found to be similar to those of the corresponding age groups in the representative sample of a German national survey conducted at the time of recruitment [11]. The study was approved by the ethics committees of the Medical Faculty of Heidelberg and the state medical board of Saarland, Germany. For the current analysis, we excluded participants with missing information on baseline weight or height, participants with BMI below 15 at any age, and those who had missing information on the underlying cause of death.

### Exposure ascertainment

This analysis used data from the baseline assessment, during which the participants completed a comprehensive questionnaire that included questions on socio-demographic characteristics, lifestyle and school education (See Additional File 1). In particular, participants were asked to recall their weight at previous ages (at ages 20, 30, 40 and 50 years). Weight and height at baseline were recorded on a standardized form by the GPs at the health check. To calculate BMI, weight (in kg) was divided by the square of height (in meters).

### Cancer ascertainment and follow-up

Information on cancer incidence (9th revision of the International Statistical Classification of Diseases, ICD-9) was obtained by record linkage with the statewide population-based Saarland Cancer Registry. Mortality follow-up was conducted by record linkage with population registries, and the underlying cause of death could be obtained from the public health authorities for 99.6% of the deceased participants. Thirteen cancer types (See Additional File 2, Supplementary Table 1) previously labeled [1] by IARC as excess weight-related cancers were included in the analysis - esophageal (adenocarcinoma), gastric (cardia), colorectal, liver, gallbladder, pancreatic, postmenopausal breast, endometrial, ovarian, kidney (renal-cell), and thyroid cancers, and multiple myeloma and meningioma. As more than 90% of women already reached menopause at the time of recruitment, most of the breast cancers diagnosed during the follow-up were postmenopausal. The current analysis included follow-up data until December 31st 2018.

### Statistical analysis

Baseline characteristics of the study populations across the quartile categories of BMI at baseline are presented using descriptive statistics.

The associations of BMI at different ages (i.e. 20, 30, 40, 50 years and at baseline) with obesity-related cancer were assessed using multivariable Cox proportional hazard models. Time to event (days) was defined as time from the enrollment to the study until (1) first obesity-related cancer diagnosis; (2) loss to follow-up; (3) death; or (4) end of follow-up, whichever came first. Two models were used. Model 1 adjusted for age and sex. Model 2 (main results) additionally adjusted for duration of school education (≤ 9, 10–11, ≥ 12 years), physical activity (inactive [< 1 h of vigorous and < 1 h of light physical activity per week], low [at least 1 h of vigorous or light physical activity, but fewer hours than in medium/high category], medium/high [≥ 2 h of vigorous and ≥ 2 h of light physical activity]), smoking (pack-years), alcohol consumption (abstainer, moderate, high, and very high– details in Table 1), 1st degree family history of any cancer (yes, no), regular NSAIDs use (yes, no), and current or previous use of hormone replacement therapy (yes, no; women only). For outcomes that included CRC, models were also adjusted for previous lower endoscopy (yes, no) and for intake of red meat (at least once per day, less than once per day), vegetables (at least once per day, less than once per day) and fruit (at least once per day, less than once per day) in the 12 months preceding recruitment. For outcomes that included postmenopausal breast cancer, models were also adjusted for history of mammography (yes, no; women only), age at menarche (years; women only), and parity (yes, no; women only). Participants were classified according to standard BMI categories (normal weight below 25; overweight, 25 to below 30; obesity, ≥ 30). Additionally, hazard ratios (HRs) and 95% CIs for an increase in BMI by one standard deviation (SD) were estimated. Dose-response relationships between BMI at different ages and obesity-related cancer risk were assessed by fitting the models using restricted cubic splines with 5 knots (5th, 25th, 50th, 75th, and 95th percentile).

**Table 1:** Baseline characteristics

Characteristic	Total	BMI at baseline			
		Quartile 1 (≤ 24.8)	Quartile 2 (> 24.8 to 27.3)	Quartile 3 (> 27.3 to 30.1)	Quartile 4 (> 30.1)
Age at baseline, years, mean (SD)	62.0 (6.6)	61.2 (6.9)	62.4 (6.7)	62.5 (6.4)	61.8 (6.5)
Sex, female, *n* (%)	5,046 (54.7)	1,501 (65.1)	1,136 (49.3)	1,111 (48.2)	1,298 (56.3)
BMI at 20, kg/m^2^, mean (SD)	22.0 (3.2)	20.8 (2.8)	21.7 (2.7)	22.3 (2.8)	23.3 (3.9)
BMI at 30, kg/m^2^, mean (SD)	23.4 (3.4)	21.6 (2.7)	22.9 (2.7)	23.8 (2.7)	25.4 (3.9)
BMI at 40, kg/m^2^, mean (SD)	24.9 (3.7)	22.2 (2.6)	24.1 (2.6)	25.4 (2.5)	27.8 (4.2)
BMI at 50, kg/m^2^, mean (SD)	26.4 (4.1)	22.7 (2.4)	25.2 (2.3)	27.0 (2.2)	30.6 (4.5)
BMI (baseline), kg/m^2^, mean (SD)	27.7 (4.4)	22.8 (1.6)	26.0 (0.7)	28.6 (0.8)	33.5 (3.4)
Weight change since age 20, kg, mean (SD)	+ 15.8 (12.6)	+ 5.5 (8.4)	+ 12.1 (7.7)	+ 17.5 (8.0)	+ 28.1 (12.9)
Smoking behavior, *n* (%)					
Never smoker	4,465 (49.8)	1,172 (52.1)	1,087 (48.2)	1,078 (48.3)	1,128 (50.5)
Former smoker	2,949 (32.9)	552 (24.5)	764 (33.9)	836 (37.5)	797 (35.7)
Current smoker	1,554 (17.3)	525 (23.3)	403 (17.9)	316 (14.2)	310 (13.9)
Education, *n* (%)					
≤9 years	6,683 (74.4)	1,517 (67.1)	1,651 (73.4)	1,722 (76.9)	1,793 (80.2)
10–11 years	1,291 (14.4)	434 (19.2)	331 (14.7)	276 (12.3)	250 (11.2)
≥12 years	1,011 (11.3)	310 (13.7)	268 (11.9)	241 (10.8)	192 (8.6)
Physical activity, *n* (%)^1^					
Inactive	1,938 (21.1)	413 (18.0)	458 (19.9)	472 (20.6)	595 (25.9)
Low	4,177 (45.4)	1,067 (46.5)	1,016 (44.2)	1,050 (45.8)	1,044 (45.4)
Medium or high	3,076 (33.5)	817 (35.6)	825 (35.9)	772 (33.7)	662 (28.8)
Alcohol consumption, *n* (%)^2^					
Abstainer	2,679 (32.7)	655 (31.9)	582 (27.8)	617 (30.3)	825 (40.9)
Moderate	5,063 (61.8)	1,263 (61.5)	1,382 (66.1)	1,306 (64.1)	1,112 (55.1)
High	457 (5.6)	137 (6.7)	127 (6.1)	112 (5.5)	81 (4.0)
Very high	124 (1.5)	32 (1.6)	32 (1.5)	30 (1.5)	30 (1.5)
Red meat consumption, *n* (%)					
At least once per day	2,808 (33.0)	586 (27.7)	695 (32.1)	736 (34.6)	790 (37.7)
Less than once per day	5,698 (67.0)	1,531 (72.3)	1,469 (67.9)	1,392 (65.4)	1,306 (62.3)
Vegetable consumption, *n* (%)					
At least once per day	3,054 (34.9)	820 (37.4)	759 (34.5)	718 (32.9)	757 (39.9)
Less than once per day	5,688 (65.1)	1,373 (62.6)	1,443 (65.5)	1,463 (67.1)	1,409 (65.1)
Fruit consumption, *n* (%)					
At least once per day	5,491 (61.6)	1,470 (65.7)	1,367 (61.1)	1,332 (59.8)	1,332 (59.8)
Less than once per day	3,420 (38.4)	768 (34.3)	870 (38.9)	895 (40.2)	887 (40.2)
1st -degree family history of cancer, *n* (%)	4,200 (46.2)	1,075 (47.1)	1,059 (46.5)	1,049 (46.3)	1,017 (44.9)
History of lower GI endoscopy, *n* (%)	2,587 (30.4)	651 (30.5)	666 (31.1)	627 (29.6)	643 (30.6)
Regular NSAIDs use, *n* (%)	1,778 (19.3)	323 (14.0)	431 (18.7)	463 (20.1)	561 (24.4)
Women only					
Age at menarche, mean (SD)	13.6 (1.7)	13.7 (1.7)	13.5 (1.6)	13.6 (1.7)	13.4 (1.8)
Had children, *n* (%)	4,494 (91.2)	1,301 (89.1)	1,020 (91.5)	1,005 (92.6)	1,168 (92.0)
Menopausal, *n* (%)	4,498 (90.6)	1,292 (87.3)	1,034 (92.3)	1,002 (91.7)	1,170 (91.9)
Ever use of HRT, *n* (%)	2,484 (53.2)	825 (58.9)	593 (55.7)	532 (52.1)	534 (45.1)
History of mammography, *n* (%)	3,796 (78.6)	1,157 (80.0)	870 (79.3)	824 (78.0)	945 (77.0)

Abbreviations: BMI = body mass index; GI = gastrointestinal; HRT = hormone replacement therapy; NSAIDs = nonsteroidal anti-inflammatory drugs; SD = standard deviation

1– “Inactive”: <1 h of vigorous and < 1 h of light physical activity per week; “Medium or high”: ≥2 h of vigorous and ≥ 2 h of light physical activity per week; “Low”: all other not classified as “Inactive” or “Medium or high”. 2– “Moderate”: women > 0–<20 and men > 0–<40 g ethanol per day. “High”: women ≥ 20-<40 and men ≥ 40-<60 g ethanol per day. “Very high”: women ≥ 40 and men ≥ 60 g ethanol per day

Next, for each participant, we calculated the weight change from age 20 until the time of enrollment. We chose the weight at age 20, as weights at age 30 and 40 showed high correlations with the weight at baseline. For evaluating the associations of weight change with the risk of obesity-related cancer, participants were classified according to quartiles of weight change. Additionally, to investigate the independent associations of weight change and baseline BMI, we fitted a model that included both weight change and BMI at baseline (Model 3).

In cancer-specific and subgroup analyses we repeated the analysis for postmenopausal breast cancer and colorectal cancer, the two most common obesity-related cancers in this cohort, and for women and men separately. To avoid the possibility of bias due to cancer-associated prediagnostic weight loss, we repeated all analyses using baseline BMI or BMI change between age 20 and baseline as exposure variable with the first 4 years of follow-up excluded. We further examined the possibility of survivor bias related to BMI at baseline or weight change since age 20 by conducting and comparing results of age-specific analyses, after dividing the cohort into 3 age groups (i.e. 50–59, 60–65, and 66 years and older).

To check the proportional hazard assumption, we plotted the Schoenfeld residuals and found no deviations. To fill in the missing values of the covariates, we performed multiple imputation by chained equations. Weights at different ages, height, and other covariates included in the analyses were used in the imputation procedure. We performed 10 iterations and generated 5 datasets (See Additional File 2, Supplementary Table 2) using the R mice() package [12]. 

All statistical analyses were conducted using R [13] version 4.3.2.

## Results

**Fig. 1:**
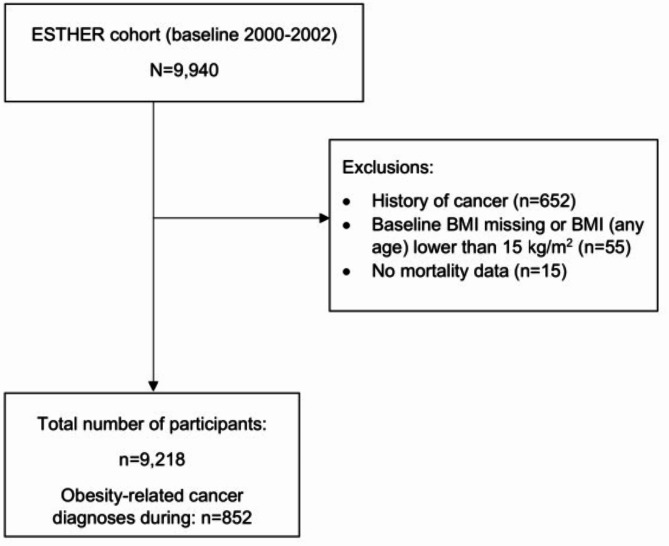
Flow chart showing the selection of the study population

The flowchart of the study population selection is shown in Fig. 1. The mean (SD) age at baseline of the 9,218 included study participants was 62.0 (6.6) years and 54.7% of participants were female. Baseline characteristics of the study population, overall and according to quartiles of BMI at baseline are presented in Table 1.

The distributions of BMI across different ages are shown in Supplementary Fig. 1 (See Additional File 2). While there was a steady increase of BMI values with age, BMI values reported for closer time points showed strong positive correlations (e.g. at age 20 and 30, or at age 50 and at baseline with correlation coefficients of about 0.80). The correlations attenuated with larger time distances (e.g. at age 20 and at baseline (mean: 62 years) with a correlation coefficient of 0.31) (See See Additional File 2, Supplementary Fig. 2).

During a median (interquartile range) follow-up of 17.1 (14.4–17.8) years, 852 participants were diagnosed with an obesity-related cancer. The associations between overweight and obesity at different ages and risk of an obesity-related cancer are shown in Table 2. Significant positive associations were found with overweight and obesity at age 50 and at baseline. Even though HRs > 1 for the association between obesity (but not overweight) and obesity-related cancer risk were consistently seen also for BMI at ages 20, 30, and 40, these associations were quite modest and none of them reached statistical significance. However, the case numbers among participants with overweight and obesity at these ages were rather small.

**Table 2:** Obesity-related cancer risk according to BMI at various ages

Time window	BMI categories (kg/m^2^)	No events	Person-years	HR (95% CI)	
				Model 1^a^	Model 2^b^
Age 20	Normal weight	765	121,706	(Reference)	(Reference)
	Overweight	74	15,509	0.79 (0.62–1.01)	0.78 (0.61–1.01)
	Obesity	13	2,032	1.15 (0.65–2.02)	1.07 (0.62–1.87)
	Per SD (3.2)	-	-	0.91 (0.84–0.99)	0.91 (0.84–0.98)
Age 30	Normal weight	658	103,778	(Reference)	(Reference)
	Overweight	164	30,832	0.90 (0.76–1.08)	0.90 (0.75–1.08)
	Obesity	30	4,637	1.14 (0.79–1.66)	1.10 (0.75–1.60)
	Per SD (3.4)	-	-	0.97 (0.91–1.05)	0.97 (0.90–1.05)
Age 40	Normal weight	503	79,277	(Reference)	(Reference)
	Overweight	281	49,966	0.98 (0.84–1.14)	0.97 (0.84–1.13)
	Obesity	68	9,961	1.28 (0.98–1.67)	1.26 (0.96–1.64)
	Per SD (3.7)	-	-	1.05 (0.98–1.12)	1.05 (0.98–1.12)
Age 50	Normal weight	316	55,596	(Reference)	(Reference)
	Overweight	392	62,821	1.20 (1.03–1.40)	1.19 (1.02–1.39)
	Obesity	144	20,830	1.42 (1.16–1.74)	1.41 (1.15–1.72)
	Per SD (4.2)	-	-	1.12 (1.05–1.19)	1.11 (1.05–1.19)
Baseline (Age between 50 and 75)	Normal weight	173	37,763	(Reference)	(Reference)
	Overweight	420	66,480	1.41 (1.18–1.68)	1.40 (1.17–1.68)
	Overweight (4y excluded)*	339	49,357	1.46 (1.19–1.78)	1.45 (1.18–1.77)
	Obesity	259	35,004	1.64 (1.35–1.99)	1.62 (1.33–1.97)
	Obesity (4y excluded)*	205	25,683	1.68 (1.36–2.09)	1.65 (1.32–2.06)
	Per SD (4.4)	-	-	1.20 (1.13–1.28)	1.20 (1.12–1.28)
	Per SD (4.4) (4y excluded)*	-	-	1.22 (1.14–1.31)	1.21 (1.13–1.30)

Abbreviations: BMI = body mass index; CI = confidence interval; HR = hazard ratio; SD = standard deviation

Normal weight corresponds to BMI below 25, overweight to BMI between 25 and < 30, and obesity to BMI above 30

^a^ Adjusted for age and sex

^b^ Adjusted for age, sex, education, previous lower endoscopy, physical activity, alcohol consumption, smoking (pack-years), 1st -degree family history of cancer, red meat intake, fruit intake, vegetable intake, current nonsteroidal anti-inflammatory drugs use, history of mammography (women) and ever use of hormone replacement therapy (women), parity (women) and age at menarche (women)

^*^ The initial 4 years of follow-up excluded

**Fig. 2:**
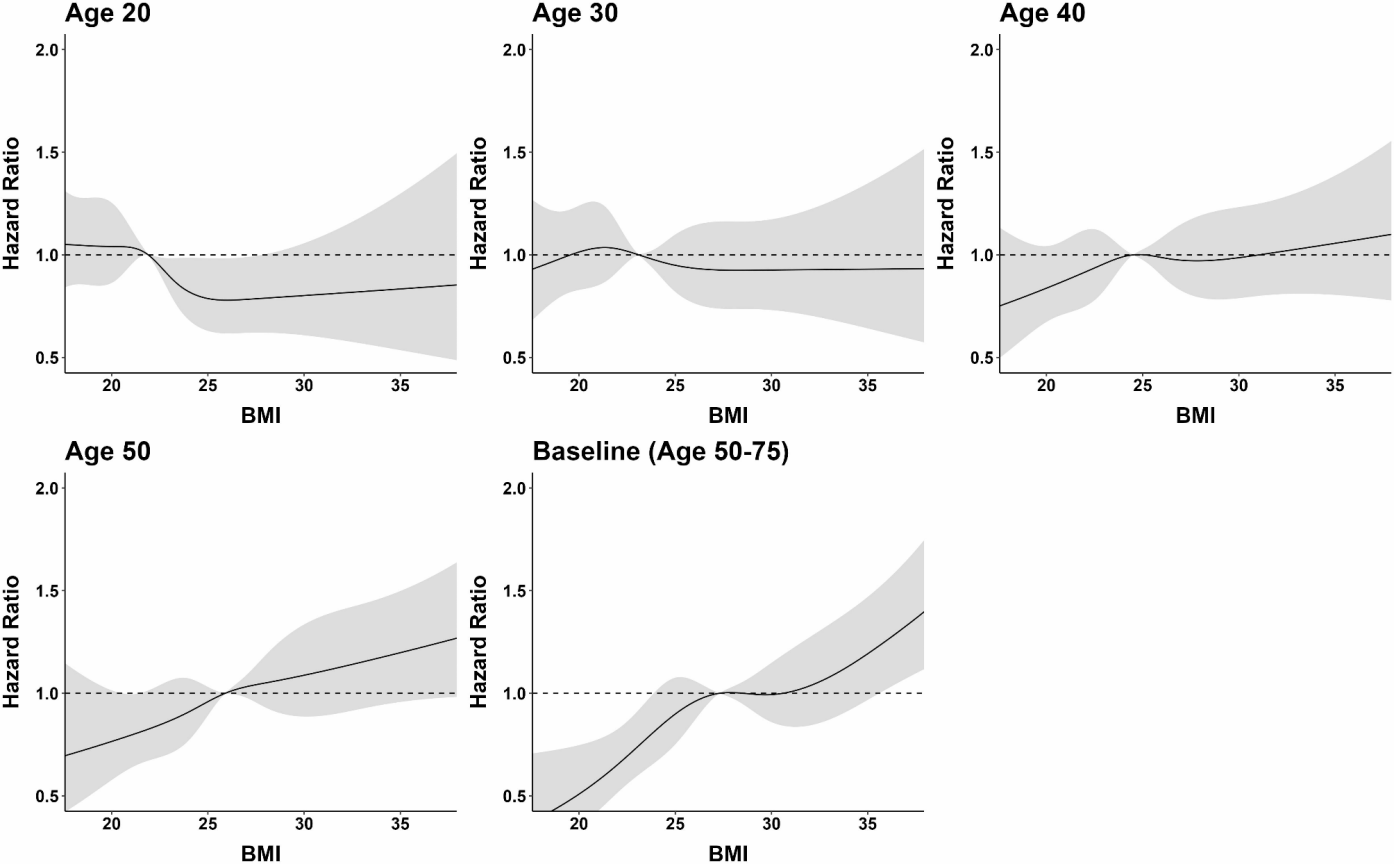
Association of BMI at different ages with obesity-related cancer risk. Restricted cubic spline regression model– knots placed at 5th, 25th, 50th, 75th, and 95th percentile, and median used as the reference. Adjusted for age, sex, education, previous lower endoscopy, physical activity, alcohol consumption, smoking (pack-years), 1st-degree family history of cancer, red meat intake, fruit intake, vegetable intake, current nonsteroidal anti-inflammatory drugs use, history of mammography (women) and ever use of hormone replacement therapy (women), parity (women) and age at menarche (women)

Figure 2 shows the dose-response relationship between BMI at different ages and obesity-related cancer risk using restricted cubic splines. No association or even inverse association between BMI up to age 40 and obesity-related cancer risk was observed, whereas an almost linear significant association was found for BMI at age 50 and at baseline.

Table 3 shows the associations of BMI at baseline and weight change since age 20 with obesity-related cancer risk. Both variables showed a clear dose-response relationship with obesity-related cancer risk. Compared to the participants who gained less than 5 kg since age 20, participants who gained > 5 to 11 kg had a 42% (95% CI 11–81%), those who gained > 11 to 18 kg had a 57% (95% CI 24–99%), and those who gained more than 18 kg had a 96% (95% CI 56–147%) increased risk of obesity-related cancer. After mutual adjustment for weight change and BMI at baseline, the estimates for BMI at baseline were attenuated to null (per SD increase– HR 1.03, 95% CI 0.91–1.16), whereas the estimates for weight change were only slightly reduced (per SD increase– HR 1.24, 95% CI 1.09–1.41).

**Table 3:** Weight change and obesity-related cancer risk

Exposure	Categories	No events	Person-years	HR (95% CI)			
				Model 1^a^	Model 2^b^	Model 2^c^	Model 3^d^
BMI at baseline (kg/m^2^)	Q1 (≤ 24.8)	166	35,389	(Reference)	(Reference)	(Reference)	(Reference)
	Q2 (> 24.8 & ≤27.3)	218	34,885	1.37 (1.11–1.67)	1.36 (1.11–1.67)	1.38 (1.10–1.74)	1.21 (0.96–1.53)
	Q3 (> 27.3 & ≤30.1)	217	35,228	1.34 (1.10–1.65)	1.33 (1.08–1.63)	1.35 (1.07–1.70)	1.07 (0.83–1.37)
	Q4 (> 30.1)	251	33,746	1.62 (1.33–1.97)	1.59 (1.31–1.95)	1.64 (1.31–2.05)	1.05 (0.78–1.41)
	Per SD (4.4)			1.20 (1.13–1.28)	1.20 (1.12–1.27)	1.21 (1.13–1.30)	1.03 (0.91–1.16)
Weight change since age 20 (kg)	Q1 ( ≤ + 5)	144	35,167	(Reference)	(Reference)	(Reference)	(Reference)
	Q2 ( > + 5 & ≤+11)	203	34,882	1.42 (1.14–1.77)	1.43 (1.14–1.78)	1.42 (1.11–1.81)	1.36 (1.06–1.75)
	Q3 ( > + 11 & ≤+18)	226	34,801	1.57 (1.27–1.93)	1.56 (1.26–1.92)	1.57 (1.24–1.99)	1.45 (1.13–1.87)
	Q4 ( > + 18)	279	34,397	1.97 (1.61–2.42)	1.96 (1.60–2.40)	1.96 (1.56–2.47)	1.66 (1.24–2.24)
	Per SD (+ 12.7)			1.27 (1.19–1.35)	1.27 (1.19–1.35)	1.27 (1.18–1.36)	1.24 (1.09–1.41)

Abbreviations: BMI = body mass index; CI = confidence interval; HR = hazard ratio; Q = quartile; SD = standard deviation

^a^ Adjusted for age and sex

^b^ Adjusted for age, sex, education, previous lower endoscopy, physical activity, alcohol consumption, smoking (pack-years), 1st -degree family history of cancer, red meat intake, fruit intake, vegetable intake, current nonsteroidal anti-inflammatory drugs use, history of mammography (women) and ever use of hormone replacement therapy (women), parity (women) and age at menarche (women)

^c^ The initial 4 years of follow-up excluded

^d^ Mutually adjusted for BMI at baseline and weight change since age 20

The associations of weight change and BMI at baseline with postmenopausal breast cancer and colorectal cancer are shown in Supplementary Table 3 (See Additional File in 2). For postmenopausal breast cancer, a non-significant negative association was found for an SD increase in BMI at age 20 (HR 0.91, 95% CI 0.80–1.04), a significant positive association for an SD increase in BMI at baseline (HR 1.18, 95% CI 1.04–1.34) and for an SD increase in weight gain since age 20 (HR 1.22, 95% 1.07–1.39). Similar associations were found for colorectal cancer– HR 0.87 (95% CI 0.74–1.01) per SD increase in BMI at age 20, HR 1.13 (95% CI 0.98–1.30) per SD increase in BMI at baseline, and a HR 1.18 (95% CU 1.03–1.36) per SD increase in weight gain.

Sex-specific associations of BMI and weight change with obesity-related cancer risk are reported in Supplementary Table 4 (See Additional File 2). Patterns of association were largely similar in women and men. The association for overweight and obesity at baseline was stronger in men than in women. In both sexes, no association with obesity-related cancer risk was seen for BMI at age 20, associations were stronger for adult weight gain than for BMI at baseline, and persisted only for adult weight gain after mutual adjustment for BMI at baseline and adult weight gain. HRs estimates for BMI at baseline and weight change were almost identical across age subgroups (See Additional File 2, Supplementary Table 5).

## Discussion

In this prospective, population-based cohort study, we found no significant association between overweight and obesity in early to middle adulthood (under 50 years) and the risk of obesity-related cancer. Conversely, a robust positive association was observed between BMI in later adulthood (50 years and over) and the risk of obesity-related cancer. Strong positive associations were also found for adulthood weight gain (from age 20 to the time of recruitment). Upon simultaneous adjustment for BMI at baseline and weight gain, the associations for BMI became non-significant, whereas the associations for weight gain remained similar. These findings indicate that the impact of excess adiposity on cancer risk varies throughout adulthood, and suggest that weight gain may serve as a better predictor of obesity-related cancer than BMI measurement at a single point in time which has been used in the vast majority of epidemiological studies reported to date.

The specific biological mechanisms that underlie the association between excess weight and cancer remain incompletely understood, although they may include chronic inflammation, insulin resistance, and altered endogenous hormone metabolism [14]. Despite this, the evidence supporting a causal relationship between adulthood overweight and obesity and cancer is extensive [1]. On the other hand, research on the association between excess weight during early life and cancer has been notably sparser. Nevertheless, excess weight before or during early adulthood (often defined as age 20–30 years) has been previously linked to elevated risk of several cancer types including colorectal, pancreatic, esophageal (adenocarcinoma), gastric (cardia) and endometrial cancer [15]. 

In our study, no significant association or even an inverse association for BMI (continuous) at ages 20, 30, and 40 years with obesity-related cancer risk was observed. Patterns were similar in women and men. These results should, however, be interpreted with caution, as the majority of participants reported having had normal weight in early and middle adulthood (e.g. >80% for age 20), making the analyses underpowered due to the small number of participants with overweight or obesity at those ages. For early adulthood obesity, we have found a positive association, albeit with wide confidence intervals due to small number of events. In women, the inverse association between BMI at age 20 years and obesity-related cancer found in our study is in concordance with previous epidemiological evidence. A 2017 meta-analysis of 24 studies found that each 5-unit increase in BMI at young age (≤ 30 years) was associated with a 14% lower risk of developing postmenopausal breast cancer risk [16]. The biological mechanisms behind this phenomenon are poorly understood and require further investigation.

Studies analyzing the association of excess adiposity and cancer risk that have incorporated life course body weight information have remained scarce. However, in the past few years there have been several studies that have investigated the relationship between cumulative exposure to excess weight and cancer risk. One approach is to calculate “overweight-years”, a measure similar to pack-years of cigarette smoking. Most studies using overweight-years have found significant positive associations with obesity-related cancers, often matching the strength of associations found in studies using BMI measured at a single point in time [4–7]. Although the overweight-years approach reflects the duration and severity of excess weight exposure, it presupposes that the risk associated with excess weight remains constant throughout adult life. Another approach is to model trajectories based on repeated body weight or size measurements. The use of weight trajectory modeling to investigate cancer risk remains limited, with only a handful of studies exploring this approach [8, 17–21]. The largest of these, conducted by Song and colleagues, compared five different body shape trajectories in terms of cancer risk [8]. In women, the risk of obesity-related cancers was highest among those with an upward trajectory, with the “lean-marked increase” group showing a 39% increased risk and the “heavy-stable/increase” group showing a 28% increase, compared to those who maintained a “lean-stable” profile. In men, larger body size at any age was associated with a nonsignificant increase in obesity-related cancer risk.

For weight gain since age 20, our study found positive associations with obesity-related cancer, somewhat stronger than those found for BMI at baseline. The associations were similar in women and in men, and similar strengths of the association were found for postmenopausal breast and colorectal cancer. To our knowledge, only one comprehensive systematic review and meta-analysis has explored the relationship between adult weight gain and the risk of obesity-related cancers. Keum et al. (2015) [9] reported significant positive associations for postmenopausal breast, endometrial and ovarian cancer, and colon (men) and kidney cancer. The magnitude of these associations was found to be stronger than those reported in previous meta-analyses considering BMI at baseline. Our findings for weight gain and postmenopausal breast cancer are consistent with previous meta-analyses [9, 16, 22]. WCRF’s 2018 report [22] and Keum et al. [9] reported summary relative risks of 1.06 (1.05–1.08) and 1.11 (1.08–1.13) for each 5 kg increase in weight gain, respectively. In alignment with our findings, a 2020 study by Renehan and colleagues [23] found weight gain (since age 20) to be a stronger postmenopausal cancer risk predictor than BMI at baseline (age 47–73 years)– HR 1.16 (1.11–1.23) and 1.05 (0.99–1.11) per SD increase in weight gain and BMI, respectively. Furthermore, Keum et al. [9] identified two studies [24, 25] in their breast cancer meta-analyses that have provided results after mutual adjustment for weight gain and current BMI. Both studies found that the positive associations with weight gain remained significant, while the associations with BMI were attenuated to null. Our study similarly observed that mutual adjustment maintained the associations of weight gain with obesity-related cancers, heightened the association with colorectal cancer, and slightly weakened the association with postmenopausal breast cancer.

Our findings, supported by the existing literature, suggest that weight gain may be a better risk predictor for obesity-related cancer risk than BMI. The observed patterns appear plausible on several grounds. First, BMI does not distinguish between lean muscle mass and adipose tissue, nor does it reflect the distribution of fat mass and fat mass gain. In contrast, adult weight gain is largely due to an increase in fat rather than lean tissue. In women, fat tends to accumulate around the waist during adulthood, whereas in adolescence, fat gains tend to be concentrated around hips and thighs (pear shape) [26]. Fat mass around the waist is associated with adverse metabolic changes and might be more detrimental for cancer risk [27]. Therefore, fat gain and harmful fat accumulation are better captured by adult weight gain. Second, the biological mechanisms underlying the association between excess body adiposity and cancer are expected to exert their influence over extended periods. Adult weight gain also partly reflects the trajectory and duration of exposure to excess weight.

Specific strengths of this study include its prospective cohort design, a long follow-up of a study population with socio-demographic characteristics similar to those of a representative German national survey, and the availability of detailed adulthood body weight history. Several limitations also deserve careful consideration. First, information on previous weights was based on self-reports. It has been previously shown that weight tends to be underreported, with the underestimation being more pronounced among older individuals, heavier individuals and among women [28, 29]. These patterns would likely lead to an underestimation of the excess weight-cancer associations. However, recent systematic reviews found good agreement between self-reported and measured body weight [30, 31]. Second, as mentioned, due to a limited number of participants with overweight and obesity in early and middle adulthood, our analyses lack the statistical power to firmly establish the relationship between BMI at these ages and cancer risk. Third, our findings on the association of BMI, particularly in early and middle adulthood, may not be applicable to younger generation populations, considering the dramatic rise in obesity prevalence since the youth of our study’s participants [2]. Fourth, due to sample size limitations, we could not perform further subgroup or cancer-specific analyses, e.g. for other types of obesity-related cancers.

In conclusion, our study indicates that excess weight may not have a uniform impact on cancer risk throughout life, highlighting the necessity for further investigation into the timing of gaining excess weight and its relationship with cancer. In line with prior research, we also found that adult weight gain could potentially better capture obesity-related cancer risk compared to a single BMI measure. While measures such as overweight-years and BMI trajectories capture more information on weight history and require more future research, a further advantage of weight gain over them is its ease of calculation and easier and more intuitive interpretation. Finally, though weight loss is generally advised for individuals with overweight and obesity, our results suggest that further avoidance of weight gain in adulthood itself may help to reduce the risk of cancers associated with excess adiposity.

## Electronic supplementary material

Below is the link to the electronic supplementary material.

Supplementary Material 1

Supplementary Material 2

## Data Availability

The data that support the findings of this study are not openly available due to confidentiality reasons.
