# Supplementary material for: Body mass index across adulthood, weight gain and cancer risk: a population-based cohort study
Source: BMC Cancer. 2025 Mar 17;25:488. doi: 10.1186/s12885-025-13855-0 (PMC11912780; doi:10.1186/s12885-025-13855-0)
Supplement: Supplementary file 2 — Supplementary Material 2 [file 12885_2025_13855_MOESM2_ESM.docx]

**Additional File 2**

**Body mass index across adulthood, weight gain and cancer risk: a population-based cohort study**

Marko Mandic, Fatemeh Safizadeh, Ben Schöttker, Bernd Holleczek, Michael Hoffmeister,

Hermann Brenner

**Supplementary Table 1. List of obesity-related cancer types included in this analysis.**

**Supplementary Table 2. Baseline characteristics of the study population before and after multiple imputation.**

**Supplementary Figure 1. Distribution of BMI at different ages.**

**Supplementary Figure 2. Correlation matrix for BMI at different ages.**

**Supplementary Table 3. BMI, weight change and post-menopausal breast and colorectal cancer risk.**

**Supplementary Table 4. Sex-specific associations - BMI, weight change and obesity-related cancer risk.**

**Supplementary Table 5. Age subgroups - BMI, weight change and obesity-related cancer risk**

**Supplementary Table 1****.** List of obesity-related cancer types included in this analysis

| Cancer type | ICD-9 code | ICD-10 code | Incident cases |
| --- | --- | --- | --- |
| Breast (postmenopausal) | 174.0-174.9 | C50.0-C50.6, C50.8, C50.9 | 285 |
| Colorectum (CRC) | 153.0-153.9, 154.0-154.1, 154.8 | C18.0- C18.9, C19, C20 | 270 |
| Endometrium | 182.0, 182.1, 182.8 | C54.1 | 43 |
| Kidney | 189.0 | C64 | 65 |
| Pancreas | 157.0-157.9 | C25.0-C25.4, C25.7-C25.9 | 81 |
| Esophagus  (adenocarcinoma) | 150.0-150.9 | C15.0-C15.5, C15.8, C15.9 | 23 |
| Ovary | 183.0 | C56 | 31 |
| Multiple myeloma | 203.0 | C90.0 | 32 |
| Liver | 155.0, 155.2 | C22.0, C22.2-C22.4, C22.7, C22.9 | 34 |
| Thyroid | 193 | C73 | 7 |
| Stomach (cardia) | 151.0 | C16.0 | 10 |
| Gallbladder | 156.0 | C23 | 8 |
| Meningioma | 192.1 | C70.0, C70.9 | 0 |
| Obesity-related (OBR) |  |  | 852 **^1^** |

Abbreviations: ICD = International Statistical Classification of Diseases.

^1^ The addition of incident cancer cases for each cancer type exceeds the total number of cases due to multiple incident cancers diagnosed on the same date for a number of participants.

**Supplementary Table 2.** Baseline characteristics of the study population before and after multiple imputation.

| Characteristic | Before imputation | After imputation | | | | |
| --- | --- | --- | --- | --- | --- | --- |
|  |  | Set 1 | Set 2 | Set 3 | Set 4 | Set 5 |
| Age at baseline, years, mean (SD) | 62.0 (6.6) | 62.0 (6.6) | 62.0 (6.6) | 62.0 (6.6) | 62.0 (6.6) | 62.0 (6.6) |
| Sex, female, n (%) | 5,046 (54.7) | 5,046 (54.7) | 5,046 (54.7) | 5,046 (54.7) | 5,046 (54.7) | 5,046 (54.7) |
| BMI at 20, kg/m^2^, mean (SD)^1^ | 22.0 (3.2) | 22.0 (3.2) | 22.1 (3.2) | 22.0 (3.2) | 22.0 (3.2) | 22.0 (3.2) |
| BMI at 30, kg/m^2^, mean (SD)^2^ | 23.4 (3.4) | 23.4 (3.4) | 23.4 (3.4) | 23.4 (3.4) | 23.4 (3.4) | 23.4 (3.4) |
| BMI at 40, kg/m^2^, mean (SD)^3^ | 24.9 (3.7) | 24.9 (3.7) | 24.9 (3.7) | 24.9 (3.7) | 24.8 (3.7) | 24.9 (3.7) |
| BMI at 50, kg/m^2^, mean (SD)^4^ | 26.4 (4.1) | 26.4 (4.2) | 26.4 (4.2) | 26.4 (4.2) | 26.4 (4.1) | 26.4 (4.2) |
| BMI (baseline), kg/m^2^, mean (SD) | 27.7 (4.4) | 27.7 (4.4) | 27.7 (4.4) | 27.7 (4.4) | 27.7 (4.4) | 27.7 (4.4) |
| Weight change since age 20, kg, mean (SD)^5^ | +15.8 (12.6) | +15.8 (12.7) | +15.8 (12.7) | +15.8 (12.7) | +15.8 (12.7) | +15.8 (12.6) |
| Smoking behavior, n (%)^6^ |  |  |  |  |  |  |
| Never smoker | 4,465 (49.8) | 4,599 (49.9) | 4,616 (50.1) | 4,600 (49.9) | 4,614 (50.1) | 4,623 (50.2) |
| Former smoker | 2,949 (32.9) | 3,017 (32.7) | 3,013 (32.7) | 3,028 (32.8) | 3,015 (32.7) | 3,011 (32.7) |
| Current smoker | 1,554 (17.3) | 1,602 (17.4) | 1,589 (17.2) | 1,590 (17.2) | 1,590 (17.2) | 1,584 (17.2) |
| Education, n (%)^7^ |  |  |  |  |  |  |
| ≤9 years | 6,683 (74.4) | 6,867 (74.5) | 6,870 (74.5) | 6,870 (74.5) | 6,866 (74.5) | 6,869 (74.5) |
| 10-11 years | 1,291 (14.4) | 1,320 (14.3) | 1,309 (14.2) | 1,316 (14.3) | 1,314 (14.3) | 1,319 (14.3) |
| ≥12 years | 1,011 (11.3) | 1,031 (11.2) | 1,039 (11.3) | 1,032 (11.2) | 1,038 (11.3) | 1,030 (11.2) |
| Physical activity, n (%)^8^ |  |  |  |  |  |  |
| Inactive | 1,938 (21.1) | 1,945 (21.1) | 1,944 (21.1) | 1,945 (21.1) | 1,944 (21.1) | 1,945 (21.1) |
| Low | 4,177 (45.4) | 4,190 (45.5) | 4,194 (45.5) | 4,191 (45.4) | 4,189 (45.4) | 4,188 (45.4) |
| Medium or high | 3,076 (33.5) | 3,083 (33.4) | 3,080 (33.4) | 3,082 (33.5) | 3,085 (33.5) | 3,085 (33.5) |
| Alcohol consumption, n (%)^9^ |  |  |  |  |  |  |
| Abstainer | 2,679 (32.7) | 3,105 (34.2) | 3,104 (34.2) | 3,118 (34.3) | 3,116 (34.3) | 3,120 (34.3) |
| Moderate | 5,063 (61.8) | 5,490 (60.4) | 5,492 (60.4) | 5,470 (60.2) | 5,479 (60.3) | 5,478 (60.3) |
| High | 457 (5.6) | 492 (5.4) | 492 (5.4) | 501 (5.5) | 493 (5.4) | 492 (5.4) |
| Very high | 124 (1.5) | 131 (1.4) | 130 (1.4) | 129 (1.4) | 130 (1.4) | 128 (1.4) |
| Red meat consumption, n (%)^10^ |  |  |  |  |  |  |
| At least once per day | 2,808 (33.0) | 3,081 (33.3) | 3,065 (33.3) | 3,057 (33.2) | 3,063 (33.2) | 3,058 (33.2) |
| Less than once per day | 5,698 (67.0) | 6,147 (66.7) | 6,153 (66.7) | 6,161 (66.8) | 6,155 (66.8) | 6,160 (66.8) |
| Vegetable consumption, n (%)^11^ |  |  |  |  |  |  |
| At least once per day | 3,054 (34.9) | 3,282 (35.6) | 3,276 (35.5) | 3,277 (35.6) | 3,284 (35.6) | 3,278 (35.6) |
| Less than once per day | 5,688 (65.1) | 5,936 (64.4) | 5,942 (64.5) | 5,941 (64.4) | 5,934 (64.4) | 5,940 (64.4) |
| Fruit consumption, n (%)^12^ |  |  |  |  |  |  |
| At least once per day | 5,491 (61.6) | 5,680 (61.6) | 5,681 (61.6) | 5,669 (61.5) | 5,682 (61.6) | 5,666 (61.5) |
| Less than once per day | 3,420 (38.4) | 3,538 (38.4) | 3,537 (38.4) | 3,549 (38.5) | 3,536 (38.4) | 3,552 (38.5) |
| 1^st^-degree family history of cancer, n (%)^13^ | 4,200 (46.2) | 4,252 (46.1) | 4,259 (46.2) | 4,267 (46.1) | 4,247 (46.1) | 4,258 (46.2) |
| History of lower GI endoscopy, n (%)^14^ | 2,587 (30.4) | 2,816 (30.6) | 2,820 (30.6) | 2,810 (30.5) | 2,819 (30.6) | 2,792 (30.3) |
| Regular NSAIDs use, n (%)^15^ | 1,778 (19.3) | 1,779 (19.3) | 1,778 (19.3) | 1,778 (19.3) | 1,779 (19.3) | 1,780 (19.3) |
| Women only |  |  |  |  |  |  |
| Age at menarche, mean (SD)^16^ | 13.6 (1.7) | 13.6 (1.7) | 13.6 (1.7) | 13.6 (1.7) | 13.6 (1.7) | 13.6 (1.7) |
| Had children, n (%)^17^ | 4,494 (91.2) | 4,600 (91.2) | 4,602 (91.2) | 4,603 (91.2) | 4,602 (91.2) | 4,594 (91.0) |
| Menopausal, n (%)^18^ | 4,498 (90.6) | 4,569 (90.5) | 4,572 (90.6) | 4,568 (90.5) | 4,571 (90.6) | 4,572 (90.6) |
| Ever use of HRT, n (%)^19^ | 2,484 (53.2) | 2,656 (52.6) | 2,688 (53.3) | 2,677 (53.1) | 2,675 (53.0) | 2,681 (53.3) |
| History of mammography, n (%)^20^ | 3,796 (78.6) | 3,949 (78.3) | 3,937 (78.0) | 3,963 (78.5) | 3,961 (78.5) | 3,946 (78.2) |

Abbreviations: BMI = body mass index; GI = gastrointestinal; HRT = hormone replacement therapy; NSAIDs = nonsteroidal anti-inflammatory drugs; SD = standard deviation.

1 – Missing value for 635 participants. 2 – Missing value for 629 participants. 3 – Missing value for 598 participants. 4 – Missing value for 473 participants. 5 – Missing value for 635 participants. 6 – Missing value for 250 participants. 7 – Missing value for 233 participants. 8 – “Inactive”: <1 h of vigorous and <1 h of light physical activity per week; "Medium or high": ≥2 h of vigorous and ≥2 h of light physical activity per week; “Low”: all other not classified as “Inactive” or “Medium or high”. Missing value for 27 participants. 9 – “Moderate”: women >0–<20 and men > 0–<40 g ethanol per day. “High”: women ≥20-<40 and men ≥40-<60 g ethanol per day. “Very high”: women ≥40 and men ≥60 g ethanol per day. Missing value for 895 participants. 10 – Missing value for 713. 11 – Missing value for 476. 12 – Missing value for 307. 13 – Missing value for 127 participants. 14 – Missing value for 721. 15 – Missing value for 6 participants. 16 – Missing value for 109. 17 – Missing value for 116. 18 – Missing value for 80 participants. 19 – Missing value for 378 participants. 20 – Missing value for 218 participants.

**
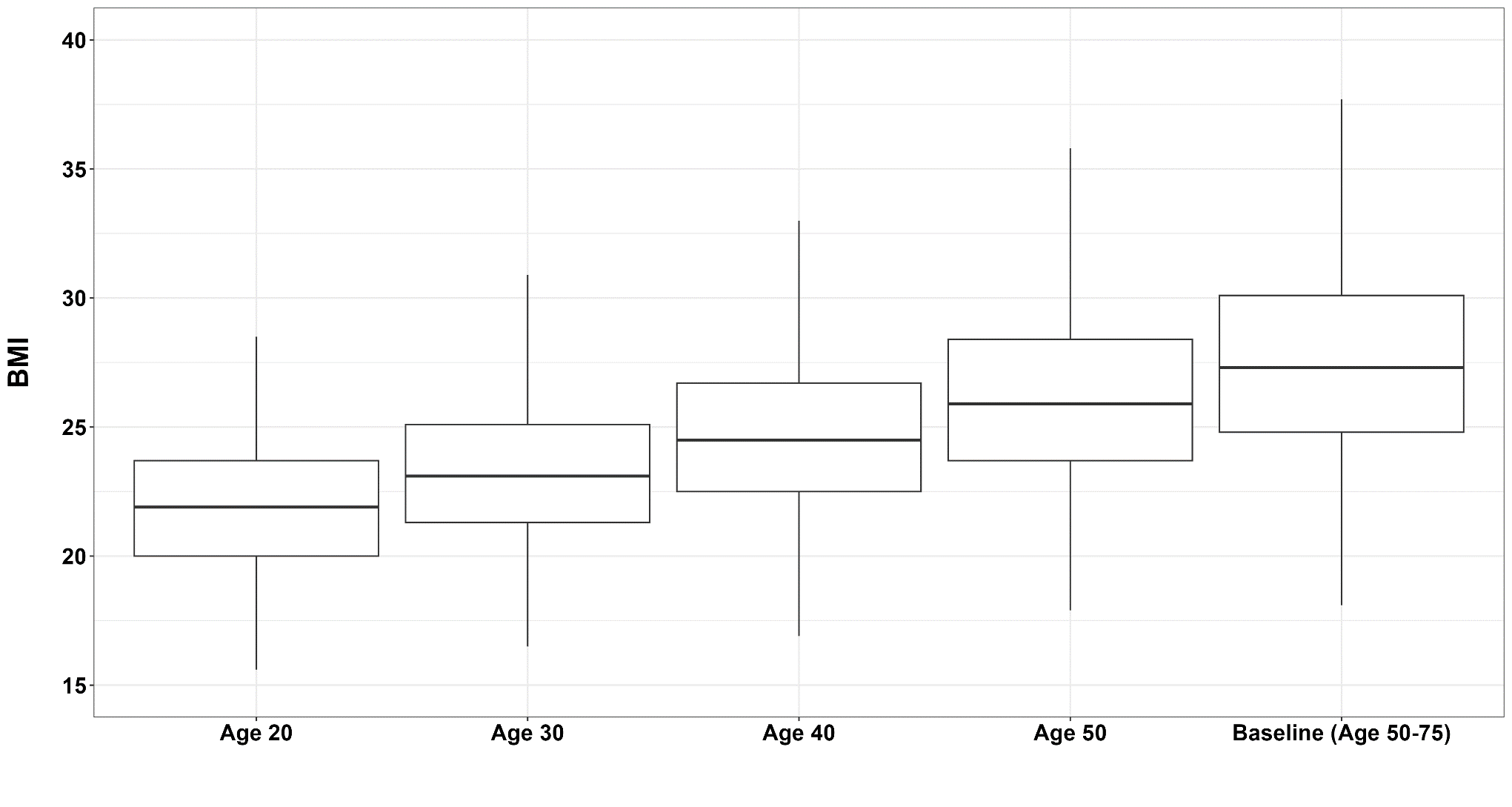
**

**Supplementary Figure 1.** Distribution of BMI at different ages. Lower and upper hinges correspond to 25th and 75th quantile of BMI, respectively; lower and upper whiskers correspond to 2.5th and 97.5th quantile, respectively. Outliers not shown.

**
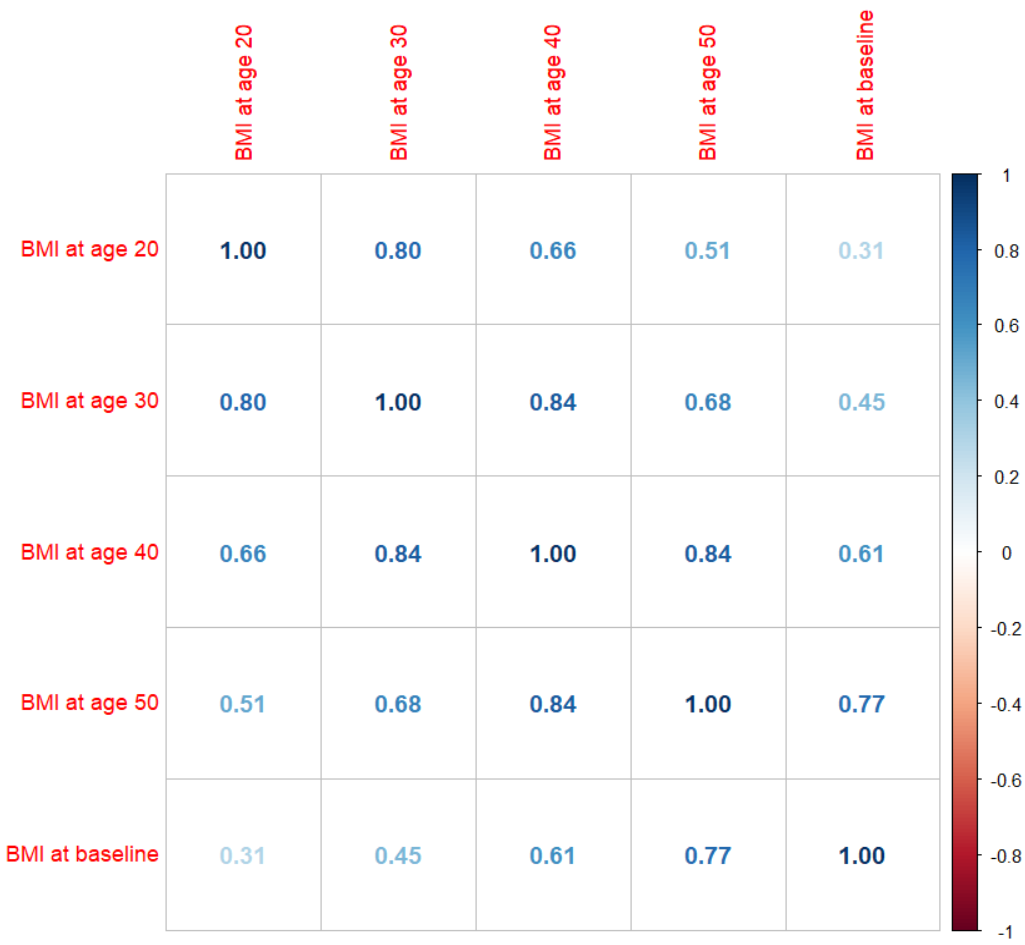
**

**Supplementary Figure 2.** Correlation matrix for BMI at different ages

**Supplementary Table 3.** BMI, weight change and post-menopausal breast and colorectal cancer risk

| Outcome | Exposure | Categories | No events | Person-years | HR (95% CI) | | | |
| --- | --- | --- | --- | --- | --- | --- | --- | --- |
|  |  |  |  |  | Model 1^a^ | Model 2^b^ | Model 2^c^ | Model 3^d^ |
| Post-menopausal  Breast cancer | BMI at age 20  (kg/m^2^) | Normal weight | 258 | 71,936 | (Reference) | (Reference) | - | - |
|  |  | Overweight | 19 | 6,327 | 0.79 (0.49-1.28) | 0.78 (0.48-1.28) | - | - |
|  |  | Obesity | 6 | 1,081 | 1.69 (0.76-3.78) | 1.59 (0.70-3.60) | - | - |
|  |  | Per SD (3.2) |  |  | 0.92 (0.80-1.05) | 0.91 (0.80-1.04) | - | - |
|  | BMI at baseline  (kg/m^2^) | Normal weight | 69 | 25,000 | (Reference) | (Reference) | (Reference) | (Reference) |
|  |  | Overweight | 137 | 33,634 | 1.48 (1.11-1.98) | 1.48 (1.11-1.99) | 1.64 (1.17-2.28) | 1.38 (0.96-1.98) |
|  |  | Obesity | 77 | 20,709 | 1.35 (0.98-1.87) | 1.38 (0.99-1.93) | 1.48 (1.01-2.17) | 0.99 (0.60-1.66) |
|  |  | Per SD (4.7) |  |  | 1.15 (1.03-1.28) | 1.16 (1.04-1.30) | 1.18 (1.04-1.34) | 1.03 (0.83-1.28) |
|  | Weight change  since age 20  (kg) | Q1 (≤+9) | 43 | 19,692 | (Reference) | (Reference) | (Reference) | (Reference) |
|  |  | Q2 (>+9 & ≤+15) | 72 | 19,958 | 1.69 (1.13-2.51) | 1.70 (1.14-2.54) | 1.83 (1.16-2.89) | 1.74 (1.09-2.78) |
|  |  | Q3 (>+15 & ≤+23) | 90 | 19,961 | 2.10 (1.43-3.08) | 2.21 (1.44-3.12) | 2.35 (1.53-3.62) | 2.13 (1.33-3.41) |
|  |  | Q4 (>+23) | 78 | 19,733 | 1.82 (1.24-2.66) | 1.88 (1.28-2.76) | 1.91 (1.22-2.99) | 1.52 (0.84-2.75) |
|  |  | Per SD (+12.5) |  |  | 1.21 (1.08-1.35) | 1.22 (1.09-1.37) | 1.22 (1.07-1.39) | 1.19 (0.95-1.49) |
| Colorectal cancer | BMI at age 20  (kg/m^2^) | Normal weight | 245 | 124,515 | (Reference) | (Reference) | - | - |
|  |  | Overweight | 21 | 15,506 | 0.63 (0.40-0.98) | 0.62 (0.39-0.97) | - | - |
|  |  | Obesity | 4 | 2,107 | 1.06 (0.39-2.86) | 1.03 (0.38-2.78) | - | - |
|  |  | Per SD (3.2) |  |  | 0.87 (0.75-1.01) | 0.87 (0.75-1.01) | - | - |
|  | BMI at baseline  (kg/m^2^) | Normal weight | 59 | 38,414 | (Reference) | (Reference) | (Reference) | (Reference) |
|  |  | Overweight | 136 | 67,966 | 1.14 (0.84-1.56) | 1.12 (0.82-1.53) | 1.09 (0.77-1.54) | 0.90 (0.62-1.31) |
|  |  | Obesity | 75 | 35,945 | 1.31 (0.93-1.84) | 1.26 (0.89-1.78) | 1.16 (0.78-1.72) | 0.74 (0.45-1.24) |
|  |  | Per SD (4.4) |  |  | 1.16 (1.03-1.31) | 1.15 (1.02-1.30) | 1.13 (0.98-1.30) | 0.95 (0.75-1.19) |
|  | Weight change  since age 20  (kg) | Q1 (≤+5) | 48 | 35,640 | (Reference) | (Reference) | (Reference) | (Reference) |
|  |  | Q2 (>+5 & ≤+11) | 72 | 35,576 | 1.41 (0.98-2.05) | 1.42 (0.98-2.07) | 1.49 (0.98-2.27) | 1.49 (0.97-2.30) |
|  |  | Q3 (>+11 & ≤+18) | 69 | 35,678 | 1.43 (0.99-2.06) | 1.40 (0.97-2.02) | 1.46 (0.96-2.22) | 1.47 (0.93-2.31) |
|  |  | Q4 (>+18) | 81 | 35,431 | 1.74 (1.22-2.49) | 1.70 (1.18-2.43) | 1.68 (1.10-2.55) | 1.69 (0.98-2.92) |
|  |  | Per SD (+12.6) |  |  | 1.24 (1.10-1.39) | 1.22 (1.08-1.37) | 1.18 (1.03-1.36) | 1.25 (0.99-1.57) |

Abbreviations: BMI = body mass index; CI = confidence interval; HR = hazard ratio; Q = quartile; SD = standard deviation.

^a^ Adjusted for age and sex.

^b^ Adjusted for age, sex, education, previous lower endoscopy (CRC only), physical activity, alcohol consumption, smoking (pack-years), 1st-degree family history of cancer, red meat, fruit and vegetable intake (CRC only), current nonsteroidal anti-inflammatory drugs use, history of mammography (women; breast cancer only) and ever use of hormone replacement therapy (women; breast cancer only), parity (women; breast cancer only) and age at menarche (women; breast cancer only).

^c^ The initial 4 years of follow-up excluded.

^d^ Mutually adjusted for BMI at baseline and weight change since age 20.

**Supplementary Table 4.** Sex-specific associations - BMI, weight change and obesity-related cancer risk

| Sex | Exposure | Categories | No events | Person-years | HR (95% CI) | | | |
| --- | --- | --- | --- | --- | --- | --- | --- | --- |
|  |  |  |  |  | Model 1^a^ | Model 2^b^ | Model 2^c^ | Model 3^d^ |
| Women | BMI at age 20  (kg/m^2^) | Normal weight | 508 | 70,844 | (Reference) | (Reference) | - | - |
|  |  | Overweight | 38 | 6,208 | 0.83 (0.58-1.17) | 0.80 (0.56-1.14) | - | - |
|  |  | Obesity | 9 | 1,042 | 1.18 (0.59-2.35) | 1.12 (0.56-2.23) | - | - |
|  |  | Per SD (3.2) |  |  | 0.91 (0.82-1.00) | 0.89 (0.81-0.99) | - | - |
|  | BMI at baseline  (kg/m^2^) | Normal weight | 133 | 24,744 | (Reference) | (Reference) | (Reference) | (Reference) |
|  |  | Overweight | 255 | 33,061 | 1.38 (1.12-1.70) | 1.37 (1.11-1.70) | 1.49 (1.17-1.90) | 1.22 (0.94-1.59) |
|  |  | Obesity | 167 | 20,291 | 1.50 (1.19-1.88) | 1.49 (1.18-1.88) | 1.63 (1.24-2.12) | 1.02 (0.71-1.46) |
|  |  | Per SD (4.7) |  |  | 1.18 (1.09-1.28) | 1.18 (1.09-1.28) | 1.23 (1.12-1.34) | 1.02 (0.87-1.19) |
|  | Weight change  since age 20  (kg) | Q1 (≤+9) | 90 | 19,495 | (Reference) | (Reference) | (Reference) | (Reference) |
|  |  | Q2 (>+9 & ≤+15) | 137 | 19,668 | 1.51 (1.15-2.00) | 1.52 (1.16-1.99) | 1.52 (1.12-2.06) | 1.46 (1.06-1.99) |
|  |  | Q3 (>+15 & ≤+23) | 154 | 19,673 | 1.68 (1.27-2.20) | 1.70 (1.30-2.22) | 1.79 (1.33-2.41) | 1.65 (1.20-2.27) |
|  |  | Q4 (>+23) | 174 | 19,259 | 1.99 (1.52-2.57) | 2.01 (1.55-2.61) | 2.07 (1.54-2.78) | 1.75 (1.18-2.58) |
|  |  | Per SD (+12.5) |  |  | 1.25 (1.15-1.36) | 1.25 (1.15-1.36) | 1.28 (1.17-1.41) | 1.26 (1.08-1.48) |
| Men | BMI at age 20  (kg/m^2^) | Normal weight | 258 | 50,948 | (Reference) | (Reference) | - | - |
|  |  | Overweight | 34 | 9,245 | 0.78 (0.53-1.15) | 0.78 (0.53-1.16) | - | - |
|  |  | Obesity | 5 | 959 | 1.29 (0.52-3.16) | 1.33 (0.54-3.25) | - | - |
|  |  | Per SD (3.2) |  |  | 0.94 (0.83-1.07) | 0.94 (0.83-1.07) | - | - |
|  | BMI at baseline  (kg/m^2^) | Normal weight | 40 | 13,020 | (Reference) | (Reference) | (Reference) | (Reference) |
|  |  | Overweight | 165 | 33,419 | 1.61 (0.91-1.87) | 1.57 (1.11-2.23) | 1.43 (0.98-2.08) | 1.24 (0.84-1.84) |
|  |  | Obesity | 92 | 14,713 | 2.14 (1.48-3.10) | 2.06 (1.41-3.00) | 1.80 (1.20-2.71) | 1.29 (0.78-2.14) |
|  |  | Per SD (4.7) |  |  | 1.27 (1.14-1.40) | 1.26 (1.13-1.39) | 1.22 (1.08-1.38) | 1.06 (0.87-1.30) |
|  | Weight change  since age 20  (kg) | Q1 (≤+8) | 53 | 15,387 | (Reference) | (Reference) | (Reference) | (Reference) |
|  |  | Q2 (>+8 & ≤+15) | 68 | 15,474 | 1.22 (0.84-1.76) | 1.21 (0.83-1.75) | 1.11 (0.73-1.67) | 1.07 (0.71-1.63) |
|  |  | Q3 (>+15 & ≤+22) | 72 | 15,502 | 1.40 (0.97-2.02) | 1.36 (0.94-1.96) | 1.30 (0.87-1.92) | 1.21 (0.79-1.83) |
|  |  | Q4 (>+22) | 104 | 14,789 | 2.07 (1.48-2.89) | 2.02 (1.45-2.83) | 1.90 (1.31-2.75) | 1.66 (1.04-2.64) |
|  |  | Per SD (+12.7) |  |  | 1.30 (1.17-1.44) | 1.29 (1.16-1.44) | 1.26 (1.11-1.43) | 1.20 (0.97-1.48) |

Abbreviations: BMI = body mass index; CI = confidence interval; HR = hazard ratio; Q = quartile; SD = standard deviation.

^a^ Adjusted for age.

^b^ Adjusted for age, sex, education, previous lower endoscopy, physical activity, alcohol consumption, smoking (pack-years), 1st-degree family history of cancer, red meat intake, fruit intake, vegetable intake, current nonsteroidal anti-inflammatory drugs use, history of mammography (women) and ever use of hormone replacement therapy (women), parity (women) and age at menarche (women).

^d^ Mutually adjusted for BMI at baseline and weight change since age 20.

|  | **Age category**  **50 to 59 years** | **Age category**  **60 to 65 years** | **Age category**  **66 years and above** |
| --- | --- | --- | --- |
| No. participants | 3,154 | 2,866 | 2,783 |
|  |  |  |  |
| **BMI at baseline** |  |  |  |
| HR (95% CI) (per kg/m^2^)^1^ | 1.04 (1.01-1.07) | 1.06 (1.03-1.08) | 1.04 (1.01-1.07) |
|  |  |  |  |
| **Weight change since age 20** |  |  |  |
| HR (95% CI) (per kg)^1^ | 1.02 (1.01-1.03) | 1.02 (1.01-1.03) | 1.02 (1.01-1.03) |
|  |  |  |  |

**Supplementary Table 5.** Age subgroups - BMI, weight change and obesity-related cancer risk

Abbreviations: BMI = body mass index; CI = confidence interval; HR = hazard ratio; SD = standard deviation.

First 4-years of follow-up excluded from all models

^1^ Adjusted for age, sex, education, previous lower endoscopy, physical activity, alcohol consumption, smoking (pack-years), 1st-degree family history of cancer, red meat intake, fruit intake, vegetable intake, current nonsteroidal anti-inflammatory drugs use, history of mammography (women) and ever use of hormone replacement therapy (women), parity (women) and age at menarche (women).
